# Prevalence and Predictors of the Use of Sunscreen Amongst Medical Students: A Multi-center Cross-sectional Study

**DOI:** 10.7759/cureus.4926

**Published:** 2019-06-17

**Authors:** Muhammad Mustafa Memon, Muzzammil Manzoor, Muhammad Moinuddin Ashrafi, Sahlish Kumar, Zaiyn Ul Haq, Simra Irfan, Zunaira Navid, Muhammad A Khan, Izza Shahid, Maheen Nisar, Shehryar Shaikh, Samran N Hassan, Vanita Motiani, Maaz S Khan

**Affiliations:** 1 Internal Medicine, Dow University of Health Sciences, Karachi, PAK; 2 Internal Medicine, Dow University of Health Science, Karachi, PAK; 3 Internal Medicine, Dow Medical College, Karachi, PAK; 4 Internal Medicine, Ziauddin University Hospital, Karachi, PAK; 5 Biochemistry, Ziauddin University Hospital, Karachi, PAK; 6 Medicine, Civil Hospital Karachi, Dow University of Health Sciences, Karachi, PAK

**Keywords:** prevalence, predictors, sunscreen, sunblock, medical students

## Abstract

Objective

Sun exposure is a primary preventable risk factor for skin cancer. Sunscreen has been shown to reduce the risk of certain skin cancers such as squamous cell carcinoma and melanoma. We aimed to assess the prevalence and predictors of sunscreen use among medical students in Karachi.

Methods

A multi-center cross-sectional study was conducted among 578 students from multiple medical universities in Karachi, Pakistan. The levels of use of sunscreen were recorded using a comprehensive questionnaire consisting of 29 questions. Descriptive statistics were used and p-values less than 0.05 calculated using the chi-square test were considered significant.

Results

A majority (n=441, 73.9%) of the participants in this study were female students. Sunscreen use was prevalent in 415 (69.5%) participants. Female students were more aware of the risk of skin cancer from extended sun exposure (n=186, 72.4%). Sunscreen use was significantly associated with gender (p<0.001) and the propensity to get sunburned easily (p=0.001). Few (n=19, 5.0%) students reported being well-versed regarding skin cancer and its risk factors. Most participants were aware of the use of sunscreen for sunburn prevention (n=473, 79.2%), though knowledge of the additional benefits of sunscreen, such as the prevention of skin cancer (n=257, 43.0%) and aging (n=199, 33.3%), was lacking.

Conclusion

There is an evident lack of knowledge of the importance of sunscreen protection among medical students, particularly regarding the prevention of skin cancer and skin aging. However, an overall positive attitude was observed regarding the use of sunscreen among female students. Medical students are an imperative part of our future healthcare system and should be adequately informed on sunscreen benefits and skin cancer prevention.

## Introduction

Skin cancer is regarded as one of the most common types of cancer globally, with an especially high incidence rate amongst fair-skinned individuals. Although the incidence rate of skin cancer is significantly lower amongst Africans, Asians and those of Latin American, or American-Indian descent, it manifests with significant mortality due to the atypical presentations of these cases [1]. Of particular concern is the fact that there have been increasing occurrences in both melanoma and non-melanoma skin cancers worldwide. In the US alone, melanoma incidence rates have doubled from 1982 to 2011, and in the absence of interventions, 112,000 new cases are projected till 2030 [2]. The International Agency for Research on Cancer (IARC) estimates a total of 428 new cases in 2018 alone, comprising a staggering 46.0% of the total number of cases in a five-year span [3].

The vast majority of melanomas (65.0-95.0%) are attributable to ultraviolet radiation (UVR) exposure, in particular, UV-A and UV-B radiation [4]. UVR has damaging effects on the skin by inducing DNA mutations, immunosuppression, and oxidative stress, hence leading to skin aging, actinic keratosis and DNA damage [5-6]. It is thought that UVR mutates the p53 tumor suppressor gene, a gene responsible for DNA repair, hence causing “expansion of mutated keratinocytes and initiation of skin cancer” [7]. Sunscreen, on the other hand, primarily consists of active ingredients that act either as UVR absorbers or as agents which reflect or scatter radiation [8]. These active ingredients range from organic to mineral compounds such as TiO2, and can reduce the risk of squamous cell carcinoma, and melanoma [9-10].

The southern city of Karachi is regarded as a cornerstone of Pakistan’s economy, with an estimated population of over 17 million people, with a gross domestic product of approximately $144 billion [11-12]. The prevention of cancer in the workforce of Karachi is therefore of tantamount importance to Pakistan’s economy as a whole. Furthermore, being located closer to the equator, the city of Karachi receives higher UVR levels, placing its denizens at more risk [13]. Medical students are exposed to harmful UV-A and UV-B radiation during transportation and whilst walking to classes or taking part in summer activities. Use of sunscreen has also been tied to factors such as gender and nationality. A study amongst European university students from 13 different countries found that 83.0% of men and 94.0% of women were sunbathed, and that sun protection use whilst sunbathing was 63.0% in men and 87.0% in women [14]. Furthermore, there has never been a study of this scope conducted within Pakistan regarding the use of sunscreen and its predictors. Thus, our study aims to fill this gap in research by uncovering such predictors and also by evaluating the frequency of sunscreen use by medical students in day-to-day work. In a developing country like Pakistan which is already plagued by multifarious problems, the increasing incidence of melanomas is an additional burden on human and socio-economic resources. Hence, the collected data and its findings could be used to subsequently create policies to combat conditions caused by UVR, and thus alleviate some of the burden placed on the Pakistani healthcare system.

## Materials and methods

Study Design and Participants

A multi-center cross-sectional study was conducted from April to May 2018 in Karachi. Using the random sampling technique, self-administered questionnaires were distributed amongst students of multiple medical universities in Karachi and a total of 578 responses were received. The participants gave informed consent in writing, and were assured that participation in the study was voluntary and anonymity would be maintained. Only students enrolled in the Bachelors of Medicine and Bachelors of Surgery (MBBS) programs of these universities were included in this study.

Questionnaire

A literature search was done for studies related to sun awareness and sunscreen use before crafting the questionnaire. The questionnaire comprised three sections with a total of 32 questions. The first section collected information on demographics: age, gender, university, and socio-economic level. The second section included a set of questions investigating skin type using the Fitzpatrick scale [15], sun exposure and sun protection habits, knowledge regarding sun exposure, sunscreen and skin cancer and whether this knowledge had any effect on their sun protection habits. In the second section, the participants were also asked what factors would encourage them to use sunscreen as an open-ended question. The third section was designed for sunscreen users and contained questions inquiring about the reason of use, the frequency of application, the type and sun protection factor (SPF) value of the sunscreen used, and on what basis it was chosen.

Statistical Analysis

The data were entered and analyzed using SPSS version 24.0 (IBM, Armonk, NY). Basic descriptive analyses were performed for all independent variables. Differences in personal preferences and habits regarding sun exposure and sun protection were analyzed using the chi-squared test. P-values less than 0.05 were taken as significant.

## Results

A total of 597 medical students participated in this study, of whom 441 (73.9%) were female participants. The mean age of participants was 20.7±1.6 years. Of the total participants, 481 (81.1%) came from a family background described as "quite well off." Regarding the Fitzpatrick scale for skin types, 215 (36.0%) had type III, 210 (35.2%) type II and 93 (15.6%) type I. Six participants (1.0%) had personal experience with skin cancer and 39 (6.5%) reported a family history of the disease (Table 1).

**Table 1:** Demographic Data of Medical Students from Three Different Universities in Karachi, Pakistan, 2018 (n=597) *Skin type was measured via the Fitzpatrick scale, as follows: Type I - Skin always burns, never tans (palest, with freckles) Type II - Skin usually burns, tans minimally Type III - Skin sometimes burns mildly, tans uniformly Type IV - Skin burns minimally, always tans well (moderate brown) Type V - Skin very rarely burns, tans very easily (dark brown) Type VI - Skin never burns (deeply pigmented dark brown to darkest brown)

Characteristics	No.	Percentage
Sex		
Male	156	26.1
Female	441	73.9
Family background		
Wealthy	64	10.8
Quite well off	481	81.1
Not very well off	42	7.1
Quite poor	6	1.0
Skin type*		
Type I	93	15.6
Type II	210	35.2
Type III	215	36.0
Type IV	57	9.5
Type V	19	3.2
Type VI	3	0.5
Family history of skin cancer		
Yes	39	6.5
No	556	93.1
Personal experience with skin cancer		
Yes	6	1.0
No	590	98.8

There was no report of sunscreen use from 179 (30.0%) participants. The majority (n=228, 38.2%) used sunscreen mainly to avoid getting tanned, while the second-largest group (n=165, 27.6%) used it to avoid getting sunburned. Most participants (n=299, 50.1%) avoided using sunscreen to prevent skin oiliness, while 212 (35.5%) participants answered that they simply forgot to apply sunscreen, and 137 (22.9%) participants said they did not use it as application was a hassle. A total of 360 (60.3%) participants stated that they liked themselves most when they were not tanned. Of these participants, 123 (20.6 %) applied sunscreen immediately before sun exposure and 183 (30.7%) only applied it during sunny months. Most of the students (n=340, 57.0%) were content to apply sunscreen only once a day, while 27 (4.5%) participants said they apply after it every few hours. Purchasing habits showed that most of the students (n=131, 21.9%) acquired a new tube of sunscreen once every year and an equal number (n=131, 21.9%) purchased it more than once a year. Overall, 415 people reported using sunscreen, giving a prevalence of 69.5% (Table 2).

**Table 2:** Attitude and Practices Regarding Sunscreen Application Amongst Medical Students

Attitudes and Practices	No.	Percentage
Do you apply sunscreen every time you go out in daylight?		
Always	77	12.9
Mostly	111	18.6
Sometimes	96	16.1
Rarely	131	21.9
Never	179	30.0
Is your sunscreen application a seasonal habit?		
Only during sunny months	183	30.7
All year round	119	19.9
Rarely ever	92	15.4
How often do you reapply it?		
No reapplication	340	57.0
Every hour	12	2.0
Every few hours	27	4.5
How often do you purchase a new tube of sunscreen		
Once a year	131	21.9
Less than once a year	126	21.1
More than once a year	131	21.9
When do you apply sunscreen		
Immediately before sun exposure	123	20.6
30 min before sun exposure	190	31.8
1 hr before sun exposure	48	8.0
2 hr before sun exposure	28	4.7
What prompts you to use sunscreen?		
Skin sensitivity/ condition	93	15.6
Doctor prescription	81	13.6
Avoid tan	228	38.2
Protection against skin cancer	80	13.4
Avoid sunburn	165	27.6
Friends/family encourage use	69	11.6
What prompts you to avoid using sunscreen?		
Getting tanned	30	5.0
Forgetting to	212	35.5
Applying is a hassle	137	22.9
Skin looks greasy/oily	299	50.1
Costly	49	8.2
It feels hotter	86	14.4
It is not effective	80	13.4
How do you like yourself most?		
When I am tanned	26	4.4
When I am not tanned	360	60.3
I do not care about my tan	176	29.5
My skin is naturally dark	34	5.7

A total of 473 (79.2%) participants were aware that sunscreen prevents sunburns, but only 257 (43.0%) of them knew that it prevents skin cancer and 199 (33.3%) knew sunscreen prevents skin aging. A total of 328 (54.9%) participants answered that sunscreen use did not reverse signs of aging. A total of 378 (63.3%) and 390 (65.3%) participants reported sun exposure to cause sunburn and tanning, respectively. Participants were least aware (n=205, 34.3%) about sun exposure’s effects on aging (Table 3).

**Table 3:** Knowledge of Sunscreen and Sun Exposure Characteristics Amongst Medical Students

Knowledge	No.	Percentage
Does sunscreen prevent sunburn?		
Yes	473	79.2
No	34	5.7
Do not know	86	14.4
Does sunscreen prevent skin cancer?		
Yes	257	43.0
No	106	17.8
Do not know	226	37.9
Does sunscreen prevent skin aging?		
Yes	199	33.3
No	153	25.6
Do not know	237	39.7
Does sunscreen reverse signs of aging?		
Yes	57	9.5
No	328	54.9
Do not know	202	33.8
What damage does sun exposure cause?		
Sunburn	378	63.3
Tanning	390	65.3
Skin aging	205	34.3
Hyperpigmentation	284	47.6
Skin cancer	308	51.6
None	5	0.8

Significantly more females were knowledgeable regarding the effectiveness of sunscreen in preventing sunburn (p=0.017) and aging (p<0.001). A significant association was also found between gender and the use of sunscreen, with females showing a much higher use of sunscreen as a protection method. Increased use of sunscreen, due to awareness of skin cancer from sun exposure, was also significantly associated with gender (Table 4).

**Table 4:** Association of Gender with Sunscreen Use and Knowledge of Effectiveness *P-values are statistically significant

	Male		Female			
Perceptions (agree with)	No.	Percentage	No.	Percentage	Total no.	P-value
Sunscreen reverses aging	19	0.33	38	0.67	57	0.379
Sunscreen prevents skin cancer	71	27.6	186	72.4	257	0.549
Sunscreen prevent sunburn	126	26.6	347	73.4	473	0.017*
Sunscreen prevents aging	47	23.6	152	76.4	199	0.007*
Use of sunscreen as sun protection method	41	12.9	277	87.1	318	0.010*
Does your skin cancer knowledge enforce sunscreen use	66	21.4	243	78.6	309	0.008*

Of 381 respondents, 19 (5.0%) marked their skin cancer knowledge as “excellent,” 115 (30.2%) as “good,” 161 (42.2%) as “average,” and 86 (22.5%) as “poor.” Of these, 151 (39.6%) used sunscreen with SPF 50 or more. The majority of these participants (n=161, 42.2%) had marked their skin cancer knowledge as “average,” while 30 (7.9%) of the participants did not believe in wearing sunscreen (Table 5).

**Table 5:** Association of Subjective Skin Cancer Knowledge with SPF Use *χ2=23.3, DF=12, p=0.025. **SPF: sun protection factor. ***BB=blemish balm, CC=color correction.

SPF** Used	Rating of Skin Cancer Knowledge				Total No. (%)
	Poor (%)	Average (%)	Good (%)	Excellent (%)	
0-20	9 (2.4)	4 (1.0)	8 (0.02)	1 (0.3)	22 (5.8)
20-50	31 (8.1)	50 (13.1)	42 (0.1)	9 (2.4)	132 (34.6)
50+	22 (5.8)	78 (20.5)	46 (12.1)	5 (1.3)	151 (39.6)
A BB or CC cream***	17 (4.5)	17 (4.5)	9 (2.4)	3 (0.8)	46 (12.1)
It does not matter	7 (1.8)	12 (3.1)	10 (2.6)	1 (0.3)	30 (7.9)
Total	86 (22.6)	161 (42.2)	115 (30.2)	19 (5.0)	381 (100)

Factors which were associated with the use of sunscreen included gender (p<0.001) and the risk of getting sunburned easily (p=0.001) as observed in Table 6. Other variables tested included skin type, length of sun exposure, time spent swimming and playing sports, time spent on social media, tan preference and a family history of skin disorders. These variables did not show a significant association with sunscreen use (Table 6).

**Table 6:** Factors Associated with Sunscreen Use Amongst Medical Students in Karachi, Pakistan *Skin type was measured via the Fitzpatrick scale, as follows: Type I - Skin always burns, never tans (palest, with freckles) Type II - Skin usually burns, tans minimally Type III - Skin sometimes burns mildly, tans uniformly Type IV - Skin burns minimally, always tans well (moderate brown) Type V - Skin very rarely burns, tans very easily (dark brown) Type VI - Skin never burns (deeply pigmented dark brown to darkest brown)

Predictors	Sunscreen Use					
	Always	Mostly	Sometimes	Rarely	Never	Total no.
Gender						
Male	5	7	28	43	72	155
Female	72	104	68	88	107	439
Skin type*						
Type I	14	22	12	23	21	90
Type II	28	41	33	42	65	209
Type III	26	41	34	46	67	214
Type IV	6	6	14	12	0	38
Type V	3	0	2	8	6	19
Type VI	0	1	1	0	1	3
Length of sun exposure						
1-5 hr	56	70	75	102	136	439
5-10 hr	19	34	18	24	37	132
>10 hr	1	5	3	4	5	18
Hours spent swimming/playing sports						
<1	62	95	75	102	143	477
1-2	12	13	17	23	27	92
2-3	2	1	2	4	0	9
3-4	0	1	0	2	3	6
4-5	0	0	1	0	1	2
>5	0	1	1	0	1	3
Hours spent on social media						
<2	13	14	13	18	34	92
2-4	24	39	27	51	62	203
4-6	23	26	29	28	47	153
6-8	13	22	19	25	24	103
>10	4	10	8	9	12	43
Skin preference						
Do not care about tan	18	24	24	46	64	176
Tanned	5	1	6	8	6	26
Not tanned	8	80	58	72	99	357
Skin is naturally dark	5	6	8	5	10	34
Ease of sunburn						
A couple hours in sun	25	40	43	47	48	203
Extended periods in sun	33	52	40	52	65	249
Never gets sunburned	18	17	13	29	65	142
Family history of skin disorders						
Yes	5	5	7	9	12	38
No	71	106	88	122	167	554

## Discussion

This cross-sectional study comprising 597 medical students sought to assess the knowledge, attitudes and practices of these students regarding sunscreen. We found varying levels of knowledge of the protective effects of sunscreen, with an overall intermediate to poor understanding among students. Less than half of the students were aware of the ability of sunscreen to prevent skin cancer and aging [16-18], while only 9.5% of participants knew that sunscreen can reverse the signs of aging [19]. Furthermore, female students were significantly more aware of the protection offered by sunscreen against sunburn and skin aging, and were compelled to utilize sunscreen due to their knowledge more than their male colleagues. This is in concert with previously published studies [20-22]. A possible explanation for this could be the more image-conscious and appearance-focused nature of women compared with men [23].

Moreover, around three-fifths of the students in our study liked themselves most when they were not tanned, which is in concordance with another Asian study [24]. Consequently, avoidance of tanning and sunburn were cited as the major reasons for sunscreen use by our cohort. This is in contrast to the study conducted by Awadh et al. [25], in which medical students cited friends and media as the main influencers of sunscreen use. Surprisingly, half of the students in our sample avoided sunscreen as they claimed it made their skin looks "greasy/oily."

The prevalence of sunscreen use was 69.5% in our population, which was slightly higher than the 61.0% and 59.0% prevalence reported by Jerkegren et al. [26] and Alberg et al. [27], respectively. The hot and humid climate of Karachi might play a part in this elevated prevalence of sunscreen use. Furthermore, data were collected during the summer months of April to May which could also contribute to the increased sunscreen use observed in our study. About half of students who reported sunscreen use applied it at least 30 minutes before sun exposure, which is recommended for adequate binding to the skin [28-29].

Given the lack of awareness regarding the protective effects of sunscreen against skin cancer and aging, medical students should be educated with the help of awareness campaigns and initiatives. Medical students represent a vital asset to society as future health care providers; hence, equipping them with adequate knowledge on sunscreen benefits would ultimately lead to increased sunscreen use among both patients and the general population.

Limitations

Our study has some limitations. Convenience sampling was used, which may introduce selection bias. Sunscreen use was self-reported by the participants and could not be verified. Questionnaires were filled during the summer months of April to May which may not reflect sunscreen use attitudes and practices throughout the year. The frequency of sunscreen use per day was not recorded and should be explored in future studies.

## Conclusions

Sunscreen use was prevalent among a significant majority of medical students. However, knowledge regarding the protective effects of sunscreen, particularly against skin cancer and aging, was observed to be deficient in our cohort. The strongest predictors of sunscreen use were observed to be prevention of tanning and sunburn. Further measures to educate medical students in regard to the benefits of sunscreen should be undertaken to ultimately increase its use.
